# State transitions across the Strep A disease spectrum: scoping review and evidence gaps

**DOI:** 10.1186/s12879-023-08888-4

**Published:** 2024-01-19

**Authors:** Prerana Parajulee, Jung-Seok Lee, Kaja Abbas, Jeffrey Cannon, Jean Louis Excler, Jerome H. Kim, Vittal Mogasale

**Affiliations:** 1 International Vaccine Institute, Seoul, Republic of Korea; 2 London School of Hygiene and Tropical Medicine, London, UK; 3 School of Tropical Medicine and Global Health, Nagasaki, Japan; 4 Telethon Kids Institute, University of Western Australia, Perth, Australia; 5 Harvard T.H. Chan School of Public Health, Boston, USA; 6 College of Natural Sciences, Seoul National University, Seoul, Republic of Korea; 7 World Health Organization, Geneva, Switzerland

**Keywords:** Group A *Streptococcus*, Strep A disease, Pharyngitis, Skin infection, Acute rheumatic fever, Acute poststreptococcal glomerulonephritis, Rheumatic heart disease, Invasive disease, State transition, Scoping review, Invasive disease

## Abstract

The spectrum of diseases caused by *Streptococcus pyogenes* (Strep A) ranges from superficial to serious life-threatening invasive infections. We conducted a scoping review of published articles between 1980 and 2021 to synthesize evidence of state transitions across the Strep A disease spectrum. We identified 175 articles reporting 262 distinct observations of Strep A disease state transitions. Among the included articles, the transition from an invasive or toxin-mediated disease state to another disease state (i.e., to recurrent ARF, RHD or death) was described 115 times (43.9% of all included transition pairs) while the transition to and from locally invasive category was the lowest (*n* = 7; 0.02%). Transitions from well to any other state was most frequently reported (49%) whereas a relatively higher number of studies (*n* = 71) reported transition from invasive disease to death. Transitions from any disease state to locally invasive, Strep A pharyngitis to invasive disease, and chronic kidney disease to death were lacking. Transitions related to severe invasive diseases were more frequently reported than superficial ones. Most evidence originated from high-income countries and there is a critical need for new studies in low- and middle-income countries to infer the state transitions across the Strep A disease spectrum in these high-burden settings.

**Supplementary Information:**

The online version contains supplementary material available at 10.1186/s12879-023-08888-4.

## Introduction

*Streptococcus pyogenes* (group A *Streptococcus* “OR” Strep A) is a major human bacterial pathogen responsible for infection and a broad disease spectrum. Strep A is ubiquitous and can colonize the skin and the throat leading to asymptomatic and symptomatic infections. Asymptomatic infections and carriage, though without any apparent signs and symptoms, can be detected through isolation of bacteria from the throat or skin [1, 2].

**Fig. 1:**
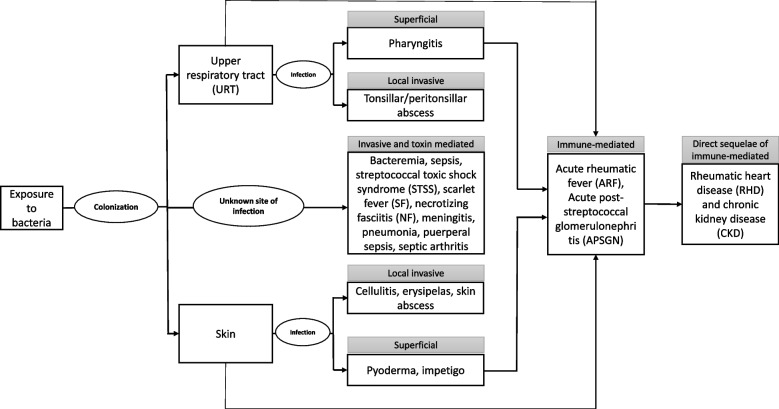
Strep A disease spectrum. Strep A disease spectrum with clinical manifestation from the time of exposure to infection, direct and indirect complications, and sequelae

We define disease state transitions as events along the pathway from being well to various Strep A-related conditions to understand the clinical manifestation and prognosis from the time of exposure to infection, direct and indirect complications, and sequelae (Fig. 1). The manifestation of symptomatic infections through the Strep A disease spectrum can range from mild, superficial infections of the throat (pharyngitis, tonsillitis) or skin (impetigo, ecthyma) to serious life-threatening invasive infection of deeper tissues and sterile sites (cellulitis, necrotizing fasciitis, osteomyelitis, etc.) [3, 4]. Both skin and throat infections may also lead to autoimmune complications after a period of latency, including acute rheumatic fever (ARF) and post-streptococcal glomerulonephritis (APSGN), and ARF may progress to rheumatic heart disease (RHD) [3, 5].

**Fig. 2:**
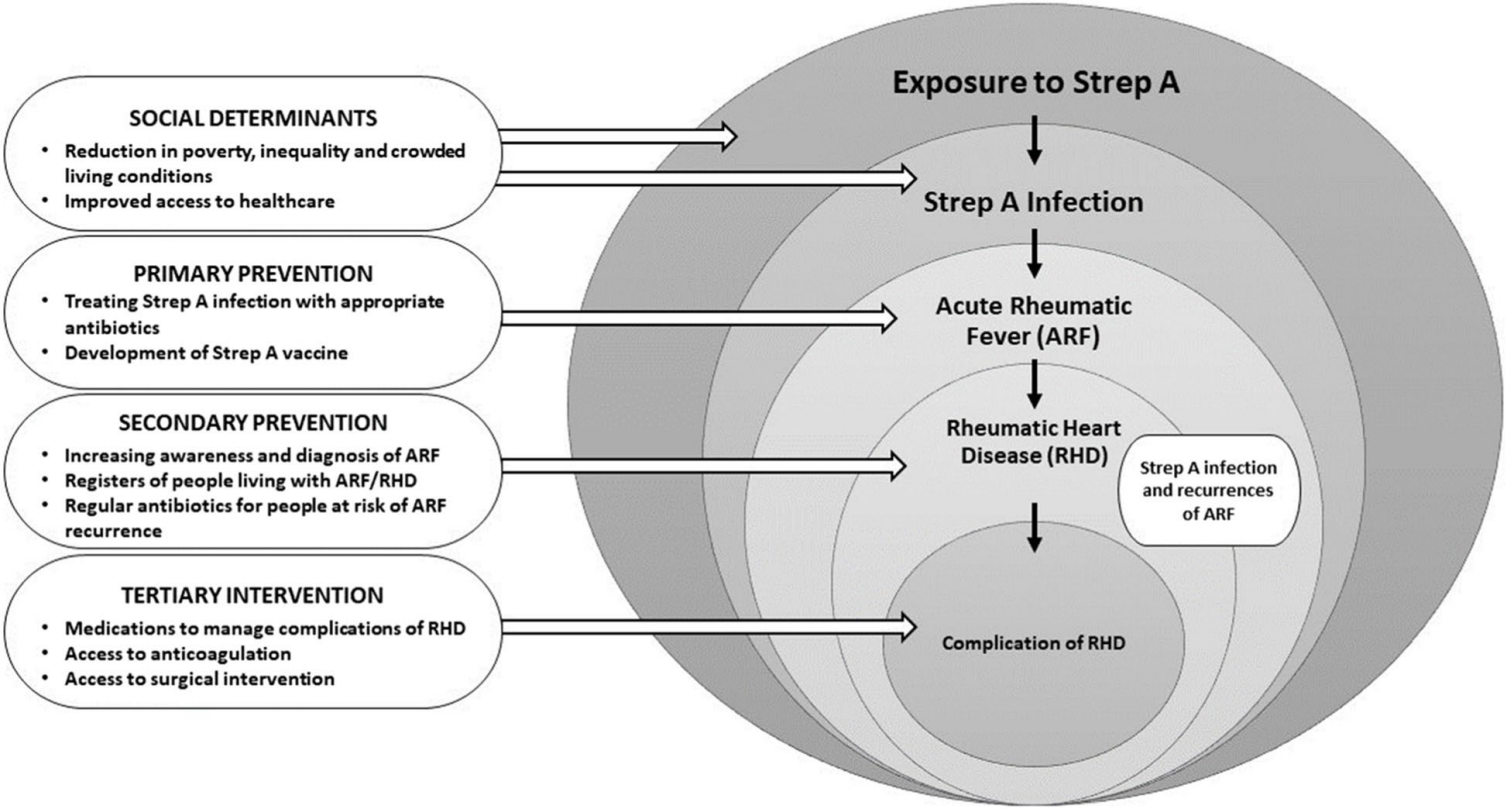
Opportunities for the prevention and management of acute rheumatic fever and rheumatic heart disease [13]. Impact of social determinants and primary, secondary, and tertiary prevention on strep A exposure, infection, and disease dynamics

The most common Strep A disease feature is pharyngitis, which occurs commonly in school-age children. Almost 15–30% of cases of all acute pharyngitis in this age group is caused by group A *Streptococcus*, with the global mean rate being 22.1 episodes per 100 child-years [6] and historically during winters and early spring in temperate climates [7]. It may be difficult to distinguish Strep A pharyngitis from other causes, but typical presentation and isolation of the bacteria assist in diagnosis. Though serological testing may be done to confirm a diagnosis, it is not useful for timely initiation of treatment as the antibody levels rise only after 2–3 weeks of infection. Tonsillar or peritonsillar abscess may follow pharyngitis [8–11]. Skin infections are the second most common Strep A disease manifestation which includes superficial infections like impetigo and pyoderma as well as invasive deep infections such as necrotizing fasciitis and cellulitis with bacteraemia [8]. The Incidence of invasive Strep A disease varies across both temporal and spatial scales and has been documented in high-income countries (HICs) [12–25] but data and evidence are limited in low- and middle-income countries (LMICs) [26, 27]. The impact of social determinants and primary, secondary, and tertiary prevention on strep A exposure, infection, and disease dynamics are illustrated in Fig. 2. Improved access to healthcare, reduction in poverty, and less crowded living conditions are associated with a lower risk of Strep A exposure and infection, while different prevention strategies are focused on averting the progression of the disease to immune-mediated complications.

The global prevalence of severe strep A disease (ARF with or without carditis, RHD, APSGN and invasive disease) is estimated to be at least 18.1 million cases and an incidence of 1.78 million new cases annually. The majority of the disease burden are attributed to RHD with at least 15.6 million cases, 282,000 new cases, and 233,000 deaths annually [8]. The prevalence (cases per 100,000 population) for RHD and APSGN are higher in LMICs and among indigenous populations [8, 9, 12]. Similarly, a higher incidence of invasive strep A bacteremia (13 cases per 100,000 person-years) in young infants, with a 25% case fatality rate among children aged under 15 years occurs in LMICs [18]. Milder infections, such as pharyngitis at 616 million cases annually, contribute to the largest number of cases to the global strep A burden [3, 8]. Although putative virulence factors of Strep A infection contributing to host–pathogen interactions have been identified, the exact mechanism that mediates the switch from localized to non-suppurative lesions like ARF or APSGN and beyond is unclear [14, 28–30]

There is an evidence gap on transitions across Strep A disease states and sequelae from exposure to serious immune-mediated complications. To address this gap, we conducted an evidence synthesis of state transitions across the Strep A disease spectrum through a scoping review.

## Methods

### Identification and selection of studies

We searched PubMed for articles published between 1980 and 2021 on state transitions across the Strep A disease spectrum, using the search criteria shown in Table 1. We conducted our search over two time periods of 01 January 1980 up to 31 November 2019 and 01 December 2019 till 31 December 2021.

**Table 1:**
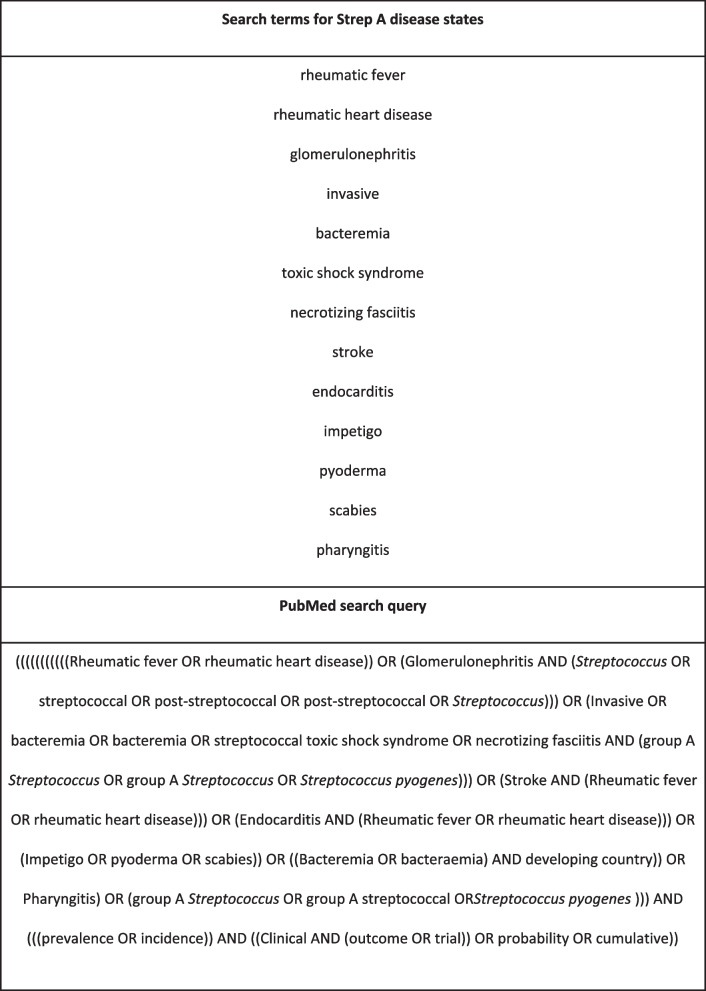
Search criteria. PubMed search query for studies on state transitions across the strep A disease spectrum

We did a two-tier screening based on predefined inclusion and exclusion criteria for initial screening and comprehensive review. During initial screening of title and abstract, we included articles defining or referencing diseases (not confirmed) that fall within the Strep A disease spectrum while we excluded articles that clearly excluded Strep A infection and disease states, such as viral pharyngitis. During comprehensive review of the full-length articles, we excluded non-English written articles, full length not available, reporting disease states other than Strep A or Strep A-associated disease sequelae, related only to epidemiology (such incidence or prevalence estimates) and treatment of Strep A disease, missing information on denominator of population under study and/or timeframe of the study, and not reporting state transition between two or more states of Strep A disease spectrum (state transition from one state to another across the disease spectrum illustrated in Fig. 1). We consulted with subject matter experts to identify additional articles missed by our search criteria.

### Data abstraction and analysis

We extracted key information from the selected studies, including title, author, year of publication, study period, geographic region (city, country), study design, sample size (total number of participants enrolled during the entire study), age category, study duration, and stages of Strep A disease spectrum (including death). We classified the income level of the countries under study according to the 2020–2021 World Bank classification for low-, lower-middle, upper-middle, and high-income countries.

Figure 1 illustrates the Strep A disease spectrum with clinical manifestation from the time of exposure to infection, direct and indirect complications, and sequelae. For this scoping we categorized the disease state in five major categories based on the framework shown in Fig. 1: 1. Superficial, 2. Locally invasive, 3. Invasive and toxin-mediated, 4. Immune-mediated, and 5. Direct sequelae of Immune-mediated. In addition to these disease categories, two more categories were included to incorporate the state pairs i.e., 1. No clinical symptoms/well as initiation state which was associated with disease transition pair and 2. Strep A related death as the absorbing state, for which transition to any other state is nil. The final disease state in each category and the disease state transition pairs are presented in Table 2.

**Table 2:** Strep A disease categories, states and transitions. Number of observations reporting different Strep A disease state transitions and incidence range. Strep A Scoping review spreadsheet

**Disease category**	**Final disease state in each category**	**Disease state transitions pairs**	**Number of studies**	**Number of observations** ^a^
No clinical symptoms / well	Well with presence of Strep A or asymptomatic Strep A infection	Well to Strep A carrier	26	31
Superficial	Strep A pharyngitis /pyoderma/ impetigo	Well to Strep A pharyngitis	8	8
		Well to Strep A skin infection	13	28^!^
		Strep A carrier to Strep A pharyngitis	-	-
		Strep A carrier to Strep A infection	-	-
Local Invasive	Tonsillar / peritonsillar abscess, cellulitis / erysipelas	Well to locally invasive	-	-
		Strep A carrier to locally invasive	-	-
		Strep A pharyngitis to locally invasive	-	-
		Strep A skin infection to locally invasive	-	-
Invasive and toxin mediated	Bacteremia, sepsis, STSS, scarlet fever, pneumonia, necrotizing fasciitis	Well to invasive Strep A	40	41
		Strep A carrier to invasive Strep A	-	-
		Throat infection toinvasive Strep A	1	1
		Strep A skin infection to invasive Strep A disease	1	1
		Locally invasive to invasive Strep A disease	3	3
Immune mediated	ARF, APSGN	Well to ARF/recurrent ARF	3	3
		Well to APSGN	5	6
		Strep A carrier to ARF	-	-
		Strep A pharyngitis to ARF	2	2
		Strep A skin infection to APSGN	1	1
		ARF to recurrent ARF	11	14
Direct sequelae of immune mediated	RHD, CKD	Well to RHD	8	9
		ARF to RHD	8	11
Strep A related death	Death	Locally invasive to death	4	4
		Non-invasive Strep A to death	1	1
		Invasive Strep A to death	70	71
		ARF to death	5	5
		Recurrent ARF to death	1	1
		RHD to death	17	21
		CKD to death	-	-

*STSS* Streptococcal toxic shock syndrome, *ARF* Acute rheumatic fever, *APSGN* Acute post-streptococcal glomerulonephritis, *RHD* Rheumatic heart disease, *CKD* Chronic kidney disease

^a^Observation: Number of state transitions across Strep A disease spectrum. In this review a total of 262 observations were inferred from 175 studies reviewed

^!^Includes 25 observations where Strep A was not identified as causative agent

## Results

### Study characteristics

**Fig. 3:**
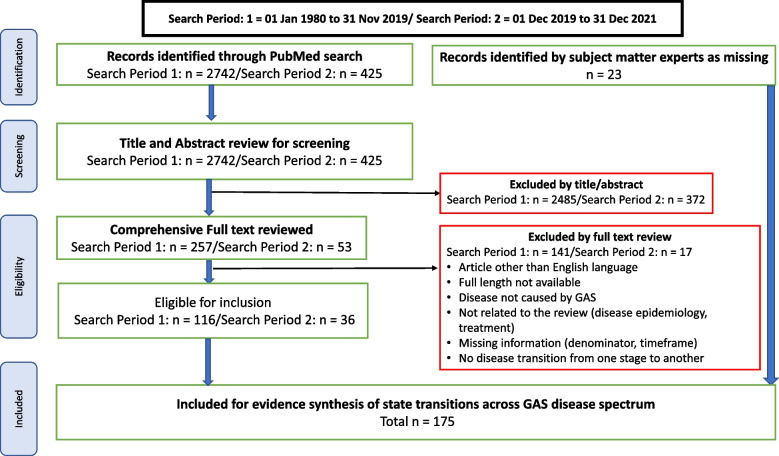
PRISMA flowchart. The flowchart illustrates the Preferred Reporting Items for Systematic Reviews and Meta Analyses (PRISMA) flow diagram of articles’ identification, screening, eligibility, and inclusion in the scoping review of strep A disease state transitions

**Fig. 4:**
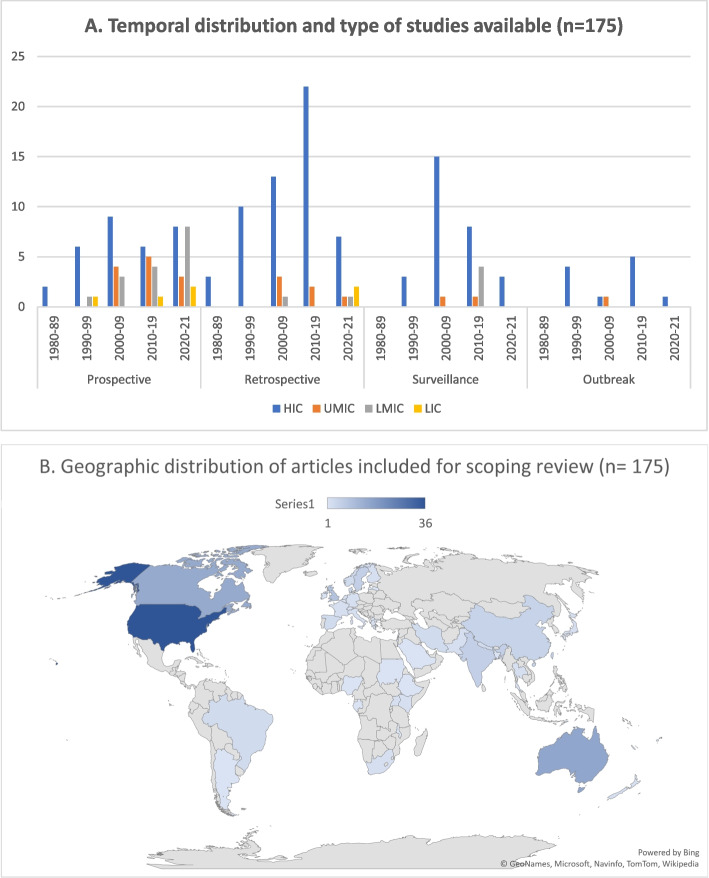
Temporal (**A**) and geographic (**B**) distribution of studies on Strep A disease transitions. (The year of publication is considered for temporal distribution of the studies reviewed and not for the initial/final date of study period)

We identified 3,167 articles based on our search criteria – 2,742 and 425 articles from the first (01 Jan 1980 to 31 Nov 2019) and the second (01 Dec 2019 to 31 Dec 2021) search periods respectively (Fig. 3). Based on title and abstract review, we excluded 2,857 articles, and we reviewed the full text of 310 articles. We found 152 articles eligible for inclusion, and 23 additional articles were provided by subject matter experts. Thereby, we conducted a scoping review of 175 articles in our evidence synthesis of disease state transitions across the strep A disease spectrum (see Figure A1 and Table A1). The majority of articles (*n* = 126, 72%) included in our scoping review were based on epidemiological data from HICs. More than half (*n* = 67/126) of these articles were only from three HICs (United States, Canada, and Australia). Only 6 articles were from low-income countries (LICs) and four articles of six were published since 2020. Forty-three articles were from middle income countries (MICs), of which 21 and 22 articles were from upper middle-income countries (UMICs) and LMICs respectively. Among the 175 articles, 65 and 63 articles were based on retrospective and prospective studies respectively, while 35 articles were based on population and laboratory-based surveillance and 12 articles on data collected during outbreaks. Figure 4 (A, B) illustrates the temporal distribution of the articles for the different types of study designs, and geographic distribution for all the 175 articles included in our scoping review.

### Strep A disease state transitions

**Fig. 5:**
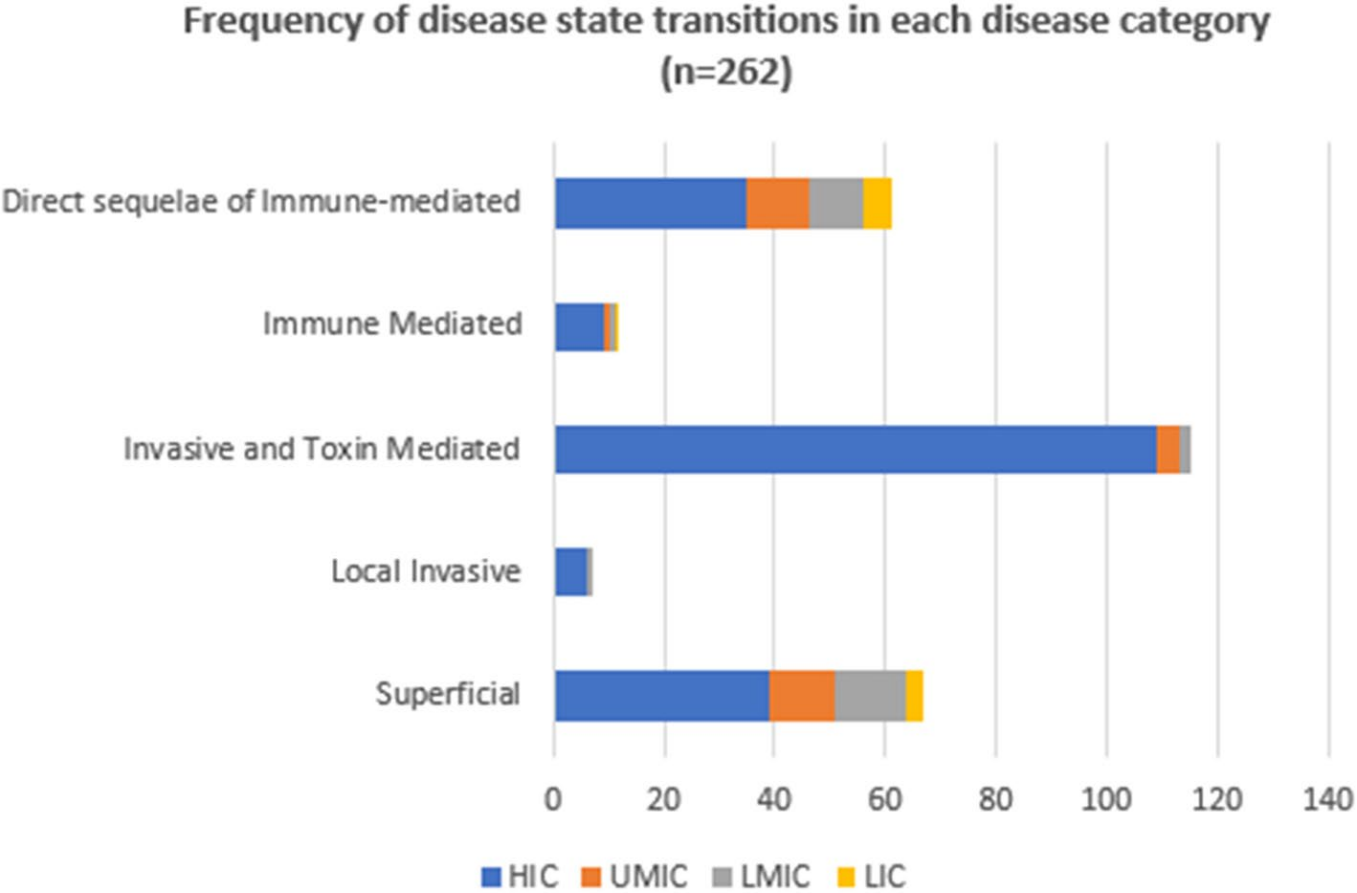
Frequency of events by strep A disease category. Strep A disease state transitions (*n* = 262) by different disease categories in low, low-middle, and upper-middle, and high-income countries

Among the 175 articles, we inferred 262 state transitions across the Strep A disease spectrum (Table 2). Figure 5 shows the frequency of Strep A disease state transitions by different disease categories in low-, middle-, and high-income countries. We could not however infer the transition rates (number of transition events from one disease state to another state for a given number of patients per unit time) between different Strep A disease states since the denominator to do this calculation, i.e., study population size, age-group, and time spent in different disease states for most of the studies could not be ascertained or was missing.

Among the included articles, the transition from an invasive or toxin-mediated disease state to another disease state (i.e., to recurrent ARF, RHD or death) was described 115 times (43.9% of all included transition pairs) while the number was low for the locally invasive category (*n* = 7). Around half (46.9%) of the Strep A disease state transition pairs were for well state (without any disease sign or symptoms) to other states, followed by invasive Strep A disease to death (27%), ARF/recurrent ARF to other states (11%), and RHD to death (8%). We focus on these state transition pairs in our evidence synthesis, while we also summarize the remaining transition pairs together.

### Well to other disease states

We identified 126 state transitions from well to seven different Strep A disease states (Table 3), of which most observations (*n* = 86) were from HICs. We classified a relatively higher number of observations focused on well to invasive Strep A disease (*n = *41), and relatively few observations (*n* = 3) for well to ARF/recurrent ARF. Among 28 of the 123 state transitions from well to other states, the age group of the study sample were less than 15 years of age.

**Table 3:** Strep A disease state transitions from well state. Reported number of observations for Strep A disease transition from the well state

**Initial state**	**Main disease state**	**Sub disease state**	**Sub disease state** (number of observations)	**Main disease state** (number of observations)
Well (*n* = 123)	APSGN			6
	ARF/Recurrent ARF			3
	Strep A Carrier			31
		Strep A Carrier (site not specified)	11	
		Strep A Skin Carrier	2	
		Strep A Throat Carrier	18	
	Skin infection			28
		Skin infection (causative agent not specified as Strep A)^a^	25	
		Strep A skin Infection	3	
	Invasive Strep A			41
	RHD			9
	Strep A Pharyngitis			8

^a^These observations are identified in studies on impetigo and scabies; however, Strep A is not explicitly identified as the causative agent

### Invasive strep A disease to death

We identified 71 state transitions from invasive Strep A disease to death. Except for two observations on Kenya and Argentina, all other observations were from HICs. Most observations are based on retrospectively collected data (*n* = 31) or surveillance studies (*n* = 19). The study team was unable to stratify according to age groups for the remaining observations due to various reasons.

### ARF/Recurrent ARF to other disease states

We identified 30 state transitions from ARF – 14 observations for ARF to recurrent ARF, 11 observations for ARF to RHD, and 5 observations for ARF to death. We identified only one observation for the state transition from recurrent ARF to death. Most observations (*n* = 21) were from the HICs (United States, Italy, Greece, Latvia, Australia, French Polynesia, and New Zealand). There were a relatively lower number of observations from other income settings of UMICs (Lebanon, Thailand, South Africa, and Brazil), LMICs (Nepal, Pakistan, and India), and LICs (Uganda).

### RHD to death

We identified 21 state transitions for RHD to death. These included 11 observations from HICs (Australia, Japan, Norway, and USA), three observations each from UMICs (China, Brazil, Thailand) and LICs (Uganda, Ethiopia and Sudan), and four observations from LMICs (India, Iran, and Timor-Leste). While five observations (three from Australia and one each from Timor-Leste and Sudan) focused on children < 16 years of age, 11 observations focused on populations > 19 years of age.

### Other strep A disease state transitions

We identified few studies with observation on other Strep A disease transitions – locally invasive to invasive Strep A disease (*n* = 3), locally invasive to death (*n* = 4), Strep A skin infection to APSGN (*n* = 1), Strep A skin infection to invasive Strep A disease (*n* = 1), Strep A Pharyngitis to ARF (*n* = 2), throat infection to invasive Strep A disease (*n* = 1), and non-invasive Strep A to death (*n* = 1).

We did not find studies for the following transitions of Strep A disease spectrum – well to locally invasive, Strep A carrier to locally invasive, Strep A pharyngitis to locally invasive, Strep A skin infection to locally invasive, Strep A pharyngitis to invasive Strep A disease, and chronic kidney disease (CKD) to death.

## Discussion

We conducted a scoping review of published articles between 1980 and 2021 to synthesize evidence of state transitions across the Strep A disease spectrum. We included 175 articles in our evidence synthesis, and we inferred 262 state transitions across the Strep A disease spectrum. There was a relatively higher number of reported transitions for the invasive and toxin-mediated category while a relatively lower number of reported transitions for the locally invasive category. While most studies focused on the transitions from well to different Strep A disease states, we also synthesized evidence on invasive Strep A disease to death, ARF/recurrent ARF to other states, and RHD to death. We identified few studies for state transitions from locally invasive to invasive Strep A disease locally invasive to death, Strep A skin infection to APSGN, Strep A skin infection to invasive Strep A disease, Strep A pharyngitis to ARF, throat infection to invasive Strep A disease, and non-invasive Strep A to death. We found no studies for state transitions from well to locally invasive, Strep A carrier to locally invasive, Strep A pharyngitis to locally invasive, Strep A skin infection to locally invasive, Strep A pharyngitis to invasive Strep A disease, and CKD to death.

Most studies were from HICs, and highlighting the paucity of evidence on epidemiological burden and transitions across the Strep A disease spectrum in LMICs with limited resources and diagnostic capacity to undertake Strep A disease surveillance and conduct observational studies to document the state transitions across the Strep A disease spectrum [31]. The current estimates of the Strep A burden of disease remain mainly derived from a limited volume of data primarily from HICs. Establishing surveillance and reporting systems in Asia, Sub-Saharan Africa, and Pacific Island Nations is therefore critically needed [32].

We inferred state transitions for invasive Strep A disease to death, and other studies have reported high mortality associated with the invasive Strep A disease [22, 33]. Acute invasive Strep A disease and its rapid progression to death may be largely preventable through timely management and focused intervention strategies to avert this transition.

We identified seven transitions from the well state to different Strep A disease states. We identified around one-fourth of the observations from the well state were to the Strep A carrier state, and reports have suggested prevalence of Strep A carriage among children to be 18% [34, 35]. While Strep A pharyngitis and pyoderma occur most frequently with an annual burden of 616 million and 111 million incident cases respectively [8], we inferred relatively higher number of studies focused on the transition from well state to invasive Strep A disease in comparison to well state to Strep A pharyngitis or Strep A skin infection. We speculate that surveillance and studies of invasive conditions were given more importance than superficial infections as there is a higher likelihood of severe health consequences, including death [36].

Our scoping review has limitations. Most articles shortlisted in this evidence synthesis are studies from hospital setting or includes highly vulnerable populations. While the original objective of our study was a systematic review of Strep A disease state transition rates, we could not infer the denominator to do this calculation. Specifically, data on study population size, age group, and time period of disease state since the onset of that state in most of the studies were missing, which precluded us from estimation of person-years of observation. Thereby, we modified the study design to a scoping review while also highlighting this evidence gap in transition rates and the need for future studies to estimate these rates. Furthermore, many of the articles which are included as evidence in this manuscript on transition from “well” to various disease syndromes, particularly invasive Strep A infections, are not evidence of transition but simple surveillance systems for the disease syndrome of interest: they only look at one of the biologic state transition “pairs”. These surveillance systems cannot and do not capture all the possible prior disease states. For example, publications based on data from invasive Strep A surveillance does not capture prior throat or skin colonization and will not differentiate between non colonized “well” state and colonized “well” state. Therefore, prospective cohort studies and population-based surveillance are critically needed to enable data collection to calculate transition rates across the Strep A disease spectrum.

Future vaccines hold potential for prevention of Strep A infection while intervention strategies including treatment with antibiotics can avert the prognosis to more serious immune-mediated complications. Further, by addressing social determinants of health, including poverty reduction and improved living conditions, and improvement in healthcare access, exposure to Strep A and early stages of Strep A disease such as pharyngitis and pyoderma could be largely reduced.

## Conclusion

We conducted a scoping review to synthesize the evidence on state transitions across the Strep A disease spectrum. Most articles included in this scoping review originated from HICs, hospital settings or includes highly vulnerable populations. Our data highlight the evidence gap in transition rates and emphasize the critical need to conduct prospective cohort and population-based surveillance studies in LMICs to infer the state transitions across the Strep A disease spectrum in these high-burden settings.

### Supplementary Information

**Additional file 1: Figure A1.** Annual distribution of articles on strep A disease state transitions during 1980-2021.** Table A1.** Articles published between 1980-2021 in low-, middle-, and high-income countries.

**Additional file 2.**

## Data Availability

The dataset analyzed during the current study is available from the corresponding author on request.

## Abbreviations

APSGN: Acute post-streptococcal glomerulonephritis
ARF: Acute Rheumatic Fever
CKD: Chronic Kidney Disease
HIC: High Income Country
LIC: Low Income Country
LMIC: Lower Middle-Income Country
RHD: Rheumatic Heart Disease
Strep A: *Streptococcus pyogenes*
STSS: Streptococcal Toxic Shock Syndrome
UMIC: Upper Middle-Income Country
