# Supplementary material for: State transitions across the Strep A disease spectrum: scoping review and evidence gaps
Source: BMC Infect Dis. 2024 Jan 19;24:108. doi: 10.1186/s12879-023-08888-4 (PMC10799450; doi:10.1186/s12879-023-08888-4)
Supplement: Supplementary file 1 — Additional file 1: Figure A1. Annual distribution of articles on strep A disease state transitions during 1980-2021. Table A1. Articles published between 1980-2021 in low-, middle-, and high-income countries. [file 12879_2023_8888_MOESM1_ESM.docx]

**Appendix:**

**Figure A1. Annual distribution of articles on strep A disease state transitions during 1980-2021.**

**Table A1. Articles published between 1980-2021 in low-, middle-, and high-income countries.**

| **Number of articles per decade per income group** | | | | | |
| --- | --- | --- | --- | --- | --- |
|  | **HIC** | **UMIC** | **LMIC** | **LIC** | **Total** |
| **1980-89** | 5 | 0 | 0 | 0 | **5** |
| **1990-99** | 23 | 0 | 1 | 1 | **25** |
| **2000-09** | 38 | 9 | 4 | 0 | **51** |
| **2010-19** | 41 | 8 | 8 | 1 | **58** |
| **2020-21** | 19 | 4 | 9 | 4 | **36** |
| **Total** | **126** | **21** | **22** | **6** | **175** |
